# HRV16 Impairs Macrophages Cytokine Response to a Secondary Bacterial Trigger

**DOI:** 10.3389/fimmu.2018.02908

**Published:** 2018-12-18

**Authors:** Jamil Jubrail, Kshanti Africano-Gomez, Floriane Herit, Engin Baturcam, Gaell Mayer, Danen Mootoosamy Cunoosamy, Nisha Kurian, Florence Niedergang

**Affiliations:** ^1^Institut Cochin, Inserm U1016, Paris, France; ^2^CNRS, UMR 8104, Paris, France; ^3^Université Paris Descartes, Sorbonne Paris Cité, Paris, France; ^4^IMED Biotech Unit, Target and Translational Science, Respiratory, Inflammation & Autoimmunity, AstraZeneca, Gothenburg, Sweden; ^5^Clinical Development, Respiratory Inhalation & Oral Development, GMD, AstraZeneca, Gothenburg, Sweden; ^6^Precision Medicine & Genomics, IMED Biotech Unit, AstraZeneca, Gothenburg, Sweden

**Keywords:** macrophage, rhinovirus, phagocytosis, cytokine, bacteria

## Abstract

Human rhinovirus is frequently seen as an upper respiratory tract infection but growing evidence proves the virus can cause lower respiratory tract infections in patients with chronic inflammatory lung diseases including chronic obstructive pulmonary disease (COPD). In addition to airway epithelial cells, macrophages are crucial for regulating inflammatory responses to viral infections. However, the response of macrophages to HRV has not been analyzed in detail. We used *in vitro* monocyte-derived human macrophages to study the cytokine secretion of macrophages in response to the virus. Our results showed that macrophages were competent at responding to HRV, as a robust cytokine response was detected. However, after subsequent exposure to non-typeable *Haemophilus influenzae* (NTHi) or to LPS, HRV-treated macrophages secreted reduced levels of pro-inflammatory or regulatory cytokines. This “paralyzed” phenotype was not mimicked if the macrophages were pre-treated with LPS or CpG instead of the virus. These results begin to deepen our understanding into why patients with COPD show HRV-induced exacerbations and why they mount a defective response toward NTHi.

## Introduction

In chronic airway inflammatory diseases such as chronic obstructive pulmonary disease (COPD) viral infections are considered a key driver for disease exacerbations. Human rhinovirus (HRV) is frequently isolated from COPD patients during exacerbations (1, 2). Although exacerbations are likely multifactorial (3), experimental rhinoviral infections in patients with COPD have been successfully utilized to understand the impact of experimental “single” infections to clinical outcomes. Patients with inflammatory airway diseases experience increased lower respiratory tract symptoms and associated fall in lung function parameters in comparison to similarly infected healthy volunteers (4, 5). Recent work in COPD patients has highlighted an increase in infections, bacterial burden and outgrowth of pathogenic bacterial species in viral infected patients (6, 7). More recently, a 2-year longitudinal follow up study (AERIS) of well-characterized COPD patients at stable state and at exacerbations reported a large increase in bacterial and viral coinfections during exacerbations (2), suggesting a possible role for viruses in regulating host defense response to bacterial infections.

Epithelial cells and innate immune cells resident in the airway lumen are key regulators of inflammation and clearance following infections. Viral infection of these cells results in abundant cytokine and chemokine release (8–13). Although epithelial cells are the primary site for HRV infections, airway macrophages are also permissive to rhinoviruses (14, 15) and regulate inflammatory responses to HRV. Furthermore, there are numerous reports of dysfunction of airway macrophages in COPD, as reviewed by Jubrail et al. (16). The emerging clinical literature of co-infections coupled with the reported bacterial clearance defects highlight the importance of dissecting cellular responses in macrophages to multi-pathogen infections and the regulation of inflammatory responses in these cells. This work therefore addresses the hypothesis that viral infections can regulate inflammatory cytokine release on subsequent bacterial infections in human macrophages.

## Materials and Methods

### Cell Culture

Human primary monocytes were isolated from the blood of healthy donors (Etablissement Français du Sang, Ile de France, Site Trinité) with the appropriate ethics prior approval as stated in the EFS/ Inserm agreement #15/EFS/012 and #18/EFS/030, ensuring that all donors gave a written informed consent, and providing anonymized samples. Density gradient sedimentation in Ficoll (GE Healthcare) was followed by adhesion on plastic at 37°C for 2 h and culture in the presence of macrophage medium (RPMI 1640 (Life Technologies) supplemented with 10% fetal calf serum (FCS) (Eurobio), 100 μg/ml penicillin/streptomycin and 2 mM L-glutamine (Invitrogen/Gibco). Monocyte-derived macrophages were then obtained as described previously (17). HeLa Ohio cells were purchased from the European Collection of Authenticated Cell Cultures (ECACC) and were cultured in DMEM GlutaMax containing 25 mM D-glucose and 1 mM sodium pyruvate (Life Technologies) supplemented with 10% FCS, 100 μg/ml penicillin/streptomycin and 2 mM L-glutamine. They were passaged every 3 days.

### Preparation of Human Rhinovirus 16 and Non-typeable *Haemophilus Influenzae*

Human rhinovirus 16 (HRV16) (VR-283, strain 11757, lot 62342987) was purchased from the American Type Culture Collection (ATCC) and stocks were produced by infecting HeLa Ohio cells as described previously (18). Briefly, supernatants from infected or mock-infected (MI) cells were collected after 48 h and clarified. In certain experimental conditions, HRV16 was inactivated with UV light (1000 mJ/cm^2^) for 20 min. Inactivation was confirmed by adding the inactivated virus to HeLa Ohio cells and checking for cytopathic effects.

NTHi strain RdKW20 (19) was purchased from the ATCC (51907). It was grown on chocolate agar plates (Biomerieux) at 37°C overnight. Bacteria were grown in LB medium supplemented with 10 μg/ml hemoglobin and 2 μg/ml β-NAD.

### Quantification of the Tissue Culture Infective Dose 50 (TCID_50_) of HRV16

HeLa Ohio cells were cultivated in 96 well plates at 1 × 10^5^ cells/well for 24 h. HRV16 was diluted 10-fold from undiluted to 10^−9^ in virus medium (DMEM GlutaMax containing 25 mM D-glucose and 1 mM sodium pyruvate supplemented with 10% FCS and 2 mM L-glutamine). Fifty microliter of each dilution was added to the cells in 8 replicate wells. Fifty microliter of virus medium was added to 2 groups of control wells in 8 replicate wells per group. Cultures were incubated for 4 days at 37°C until cytopathic effect was observed in 50% of wells. TCID_50_ was calculated using the Spearman-Karber formula as previously outlined (18).

### HRV16 and NTHi/LPS Infection of Human Macrophages

Macrophages were washed once in PBS and rested in virus medium. HRV16, HRV16^UV^ or MI supernatants were added to the macrophages and placed at room temperature for 1 h with agitation to achieve a TCID_50_ of 1 × 10^7^/ml. Cultures were then washed with virus medium and rested in macrophage medium for 24, 48 or 72 h. Prior to bacterial infection or LPS (Sigma) treatment, culture supernatants were collected and stored at −80°C for further analysis.

NTHi was grown until mid-log growth phase, centrifuged at 1692 *x g* for 5 min and resuspended in 1 ml phagocytosis medium (RPMI supplemented with 2 mM L-glutamine). NTHi was added to macrophages pre-treated with HRV16, HRV16^UV^ or MI to achieve a multiplicity of infection (MOI) of 10/cell. Cultures were then centrifuged at 602 *x g* for 2 min and placed at 37°C, 5% CO_2_ for 2 h. Alternatively, LPS was added to macrophages at a concentration of 10 ng/ml. After centrifugation, cultures were placed at 37°C, 5% CO_2_ for 2 h. At this time point, supernatants were collected and stored at −80°C for further analysis.

### Lipopolysaccharide and CpG Stimulation of Human Macrophages

Macrophages were washed once in PBS and stimulated with 10 ng/ml LPS or 0.6 μM CpG in macrophage complete medium for 24, 48 or 72 h. At each time point cultures were washed with PBS and stimulated with NTHi as listed above.

### Cytotoxicity Assay

Cytotoxicity has been measured by detection of Lactate Dehydrogenase (LDH) released in the cell supernatant with the Cytotoxicity Assay Kit according to the manufacturer's instructions (Pierce).

### Analysis of Cytokine Production Using Meso Scale Discovery®

Cytokine production by macrophages was analyzed using the Meso Scale Discovery® technology according to the manufacturer's instructions.

### Statistical Analysis

Statistical tests were performed using Graphpad prism® version 6 software. All statistical tests are listed in the figure legends and significance was determined if *p* < 0.05.

## Results

### HRV16 Infection Induces Robust Cytokine Production From Human Macrophages

**Figure 1:**
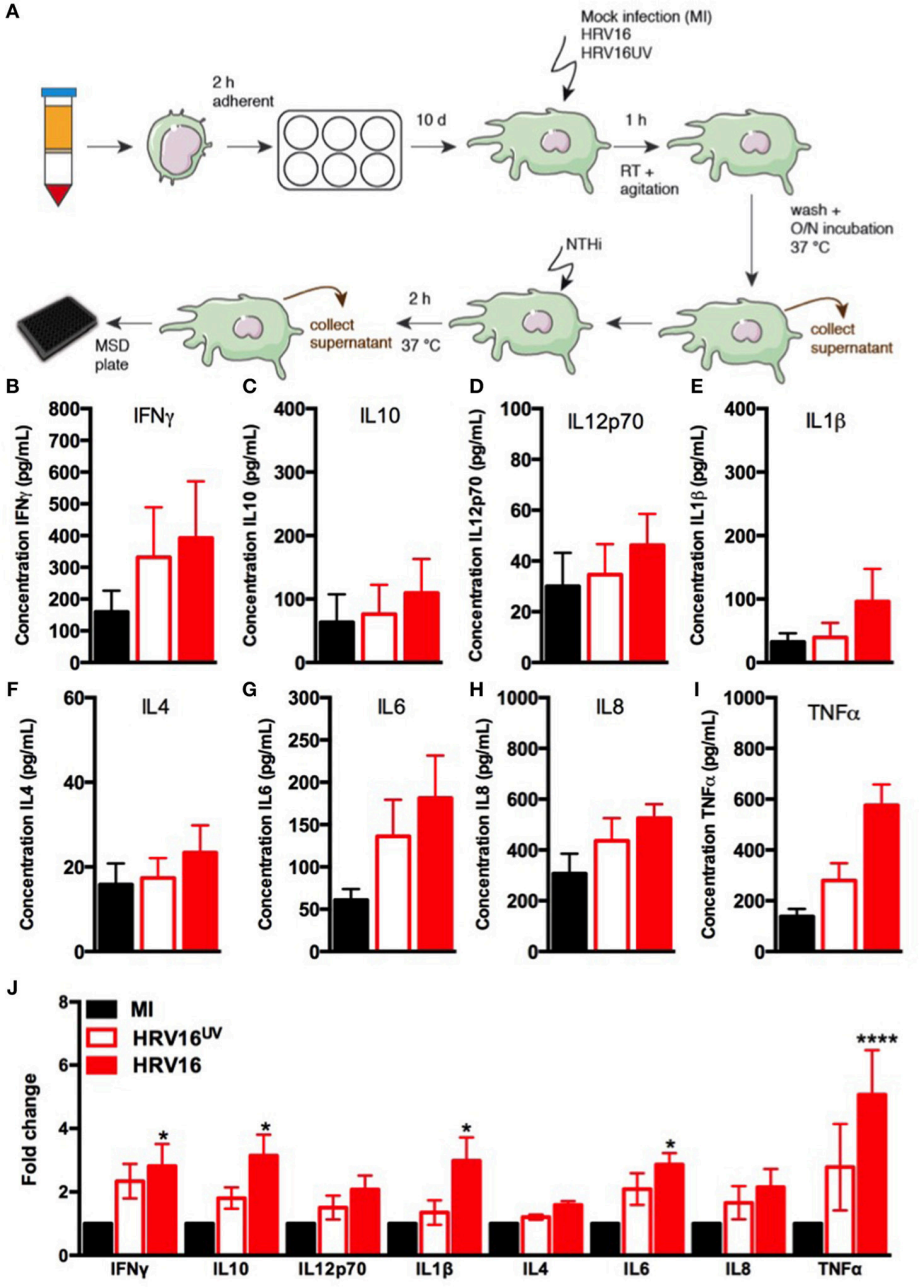
Human macrophages release pro-inflammatory and regulatory cytokines in response to HRV16. **(A)** Experimental protocol: human macrophages differentiated from blood monocytes were exposed to HRV16 (red bars), HRV16^UV^ (open bars) or MI (black bars) for 1 h and rested overnight. Supernatants were collected and analyzed by MSD. MSD results for **(B)** IFNγ, **(C)** IL10, **(D)** IL12p70, **(E)** IL1β, **(F)** IL4, **(G)** IL6, **(H)** IL8, **(I)** TNFα, **(J)** Relative fold changes for cytokine production in HRV16 and HRV16^UV^ exposed human macrophages vs. MI. *n* = 5 independent experiments on different donors. Error bars represent standard error of the mean (SEM). *p < 0.05, ****p < 0.0001 Two Way Anova with Dunnett's Post Test vs. MI.

In order to assess the ability of macrophages to respond to HRV, we challenged them with HRV16, HRV16^UV^ or MI as controls, for 1 h at room temperature followed by an overnight rest. Supernatants from virus-treated or control macrophages were analyzed by MSD to detect cytokine secretion (Figure 1A). We found that HRV16 infected macrophages produced pro-inflammatory and regulatory cytokines at 24 h (Figures 1B–J). When we analyzed the fold changes in comparison to the MI control (Figure 1J), we observed that IFNγ, IL12p70, IL4, IL6, and IL8 were produced to a similar level by HRV16 and HRV16^UV^. For IL10, IL1β, and TNFα, however, there was a trend toward more secretion after treatment with HRV16 vs. HRV16^UV^ and significantly more secretion vs. MI (Figure 1J). These results demonstrate that macrophages are competent to respond to HRV16 and effectively secrete cytokines in response to HRV.

**Figure 2:**
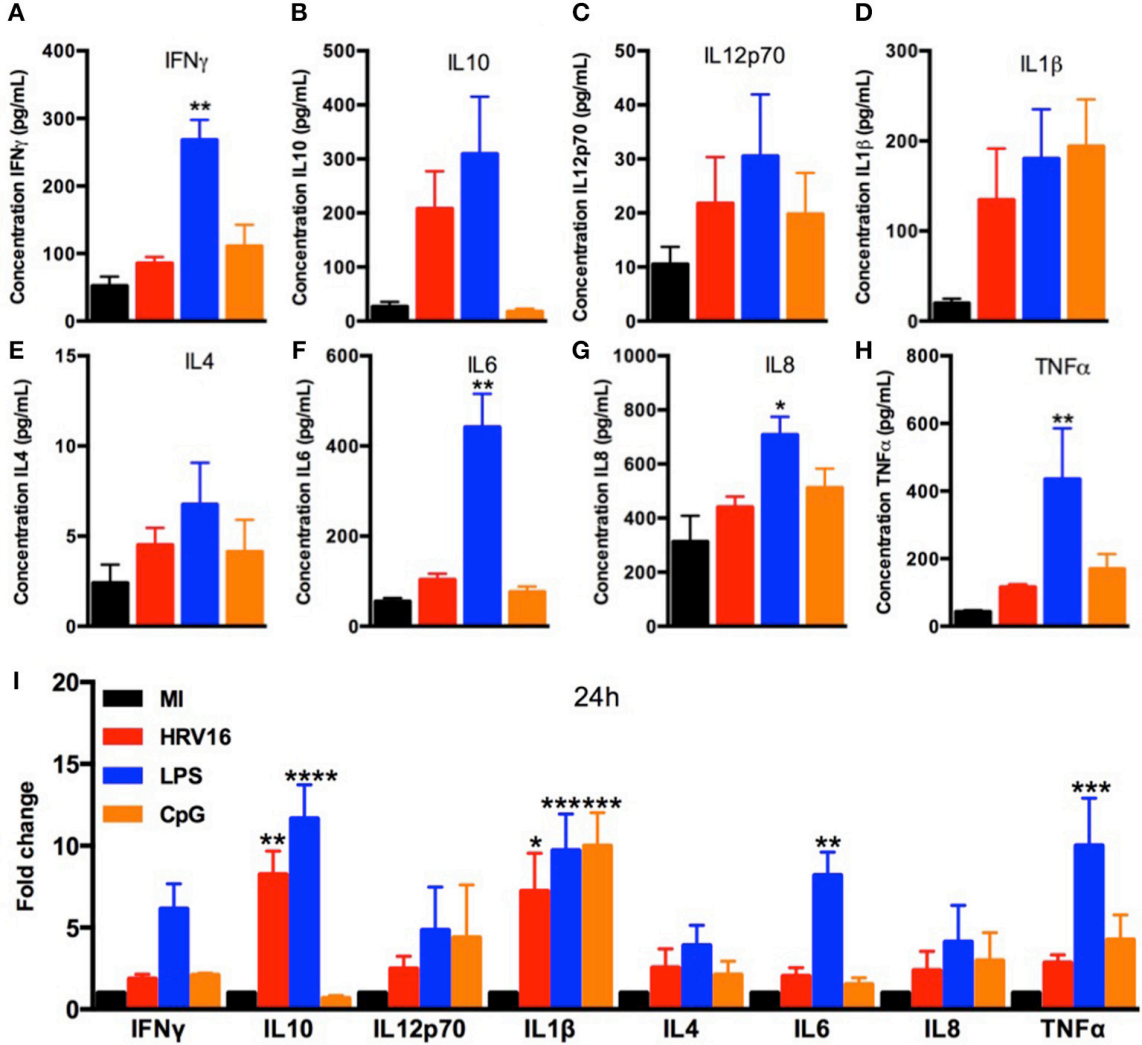
Human macrophages secrete pro-inflammatory and regulatory cytokines in response to LPS and CpG. Human macrophages were exposed to HRV16 (red bars), LPS (blue bars), CpG (orange bars) or MI (black bars) for 1 h and rested overnight. Supernatants were collected and analyzed by MSD. MSD results for **(A)** IFNγ, **(B)** IL10, **(C)** IL12p70, **(D)** IL1β, **(E)** IL4, **(F)** IL6, **(G)** IL8, **(H)** TNFα. *p < 0.5, **p < 0.01 Kruskal Wallis Test with Dunn's Post Test vs. MI. **(I)** Relative fold changes for cytokine production in HRV16, LPS and CpG exposed human macrophages vs. MI. **p < 0.01, ***p < 0.001, ****p < 0.0001 Two Way Anova with Dunnett's Post Test vs. MI. Error bars represent standard error of the mean (SEM). *n* = 4 independent experiments on different donors.

We next wanted to compare cytokine secretion in response to HRV16 to other known stimuli such as the TLR agonists LPS and CpG. For this, macrophages were treated with HRV16, LPS or CpG for 1 h and then rested overnight. We found that LPS stimulation led to increased production of all cytokines tested (Figures 2A–I), with a significant difference for IL10, IL1β, IL6, and TNFα compared to control (MI) macrophages (Figure 2I). CpG stimulation also led to cytokine production with the exception of IL10 and IL6 (Figure 2A–I). These results demonstrate that the HRV16 induced cytokine responses are similar to potent TLR macrophages activators.

### HRV16 Infection Impairs Cytokine Secretion From Human Macrophages in Response to NTHi

**Figure 3:**
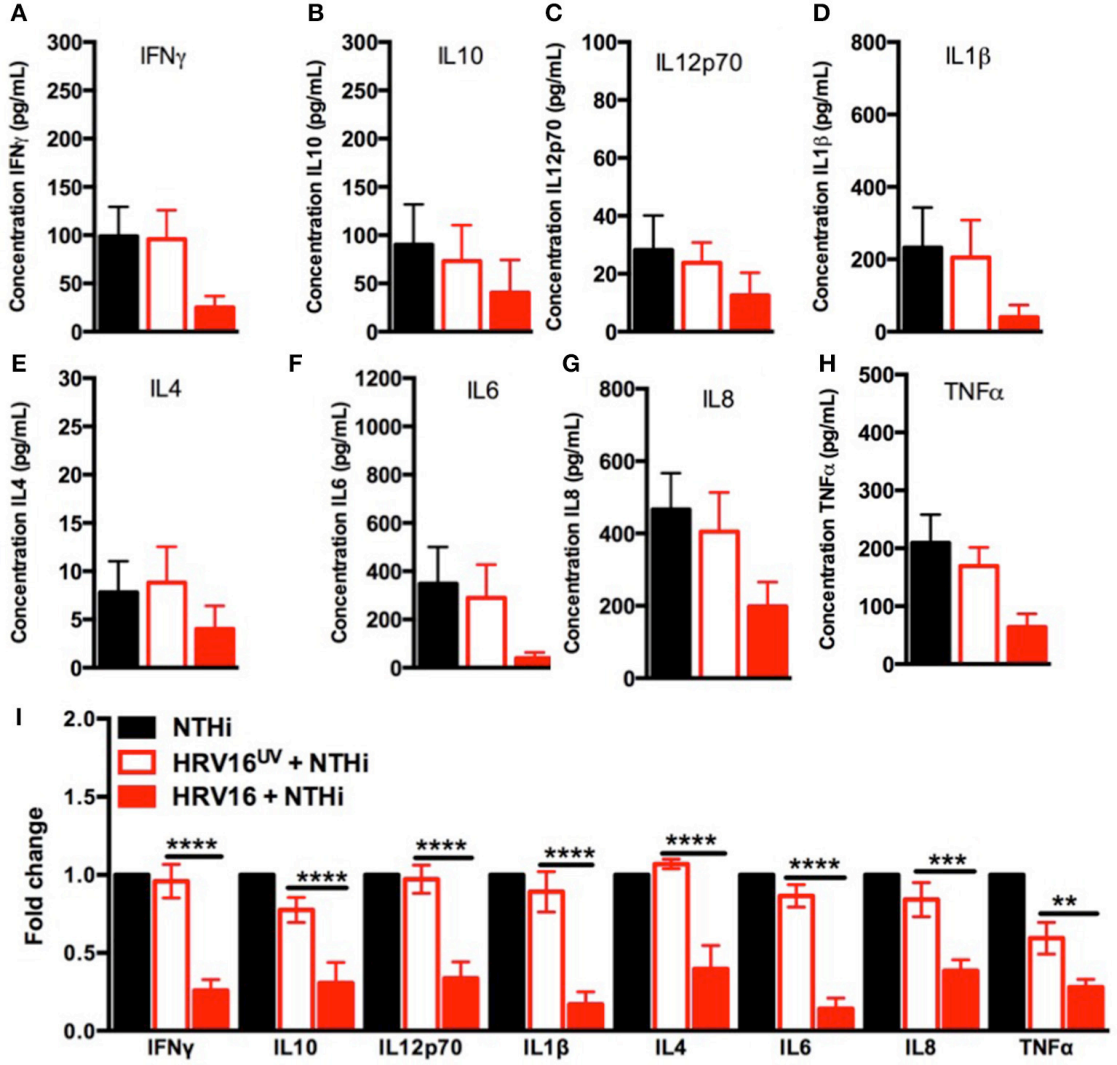
Human macrophages exposed to HRV16 cannot secrete elevated levels of pro-inflammatory and regulatory cytokines in response to NTHi. Human macrophages were exposed to HRV16 (red bars), HRV16^UV^ (open bars) or MI (black bars) for 1 h and rested overnight before being exposed to NTHi for 2 h. Supernatants were collected and analyzed by MSD. MSD results for **(A)** IFNγ, **(B)** IL10, **(C)** IL12p70, **(D)** IL1β, **(E)** IL4, **(F)** IL6, **(G)** IL8, **(H)** TNFα. **(I)** Relative fold changes for cytokine production in HRV16 and HRV16^UV^ exposed human macrophages *vs* NTHi. **p < 0.01, ***p < 0.001, ****p < 0.0001 Two Way Anova with Dunnett's Post Test vs. HRV16^UV^. Error bars represent standard error of the mean (SEM). *n* = 5 independent experiments on different donors.

We next assessed the ability of macrophages to respond to a secondary bacterial trigger. Macrophages were first treated with HRV16 or controls and were challenged 24 h later with NTHi for 2 h. Supernatants were collected and analyzed for cytokine secretion by the MSD technology (Figure 1A). We found that HRV16 exposed macrophages were unable to secrete pro-inflammatory and regulatory cytokines in response to NTHi (Figures 3A–I). There was a diminished production of all cytokines analyzed in macrophages exposed to HRV16 (Figure 3A–I) as compared with HRV16^UV^ or MI, with the greatest decreases seen for IL1β and IL6 (Figures 3D,F,I). It is interesting to note that for all cytokines analyzed, HRV16^UV^ + NTHi and MI + NTHi exposed macrophages had a similar response (Figures 3A–I). Analysis of the fold changes as compared with MI in these experiments more clearly demonstrated that HRV16 infected macrophages showed significantly diminished cytokine production in response to NTHi (Figure 3I). In contrast, despite HRV16 and HRV16^UV^ exposed macrophages showing slightly similar results for some cytokines in Figure 1 (IFNγ, IL12p70, IL6, and IL8) (Figure 1I), HRV16 exposed macrophages showed a significant reduction in all cytokine secretion in response to the bacteria (Figure 3I). This suggests that there is some regulation by the live virus and that HRV16 exposed macrophages are unable to mount an efficient response toward secondary bacterial targets.

### LPS and CpG Stimulation of Human Macrophages Does Not Impair Secondary Responses to NTHi

**Figure 4:**
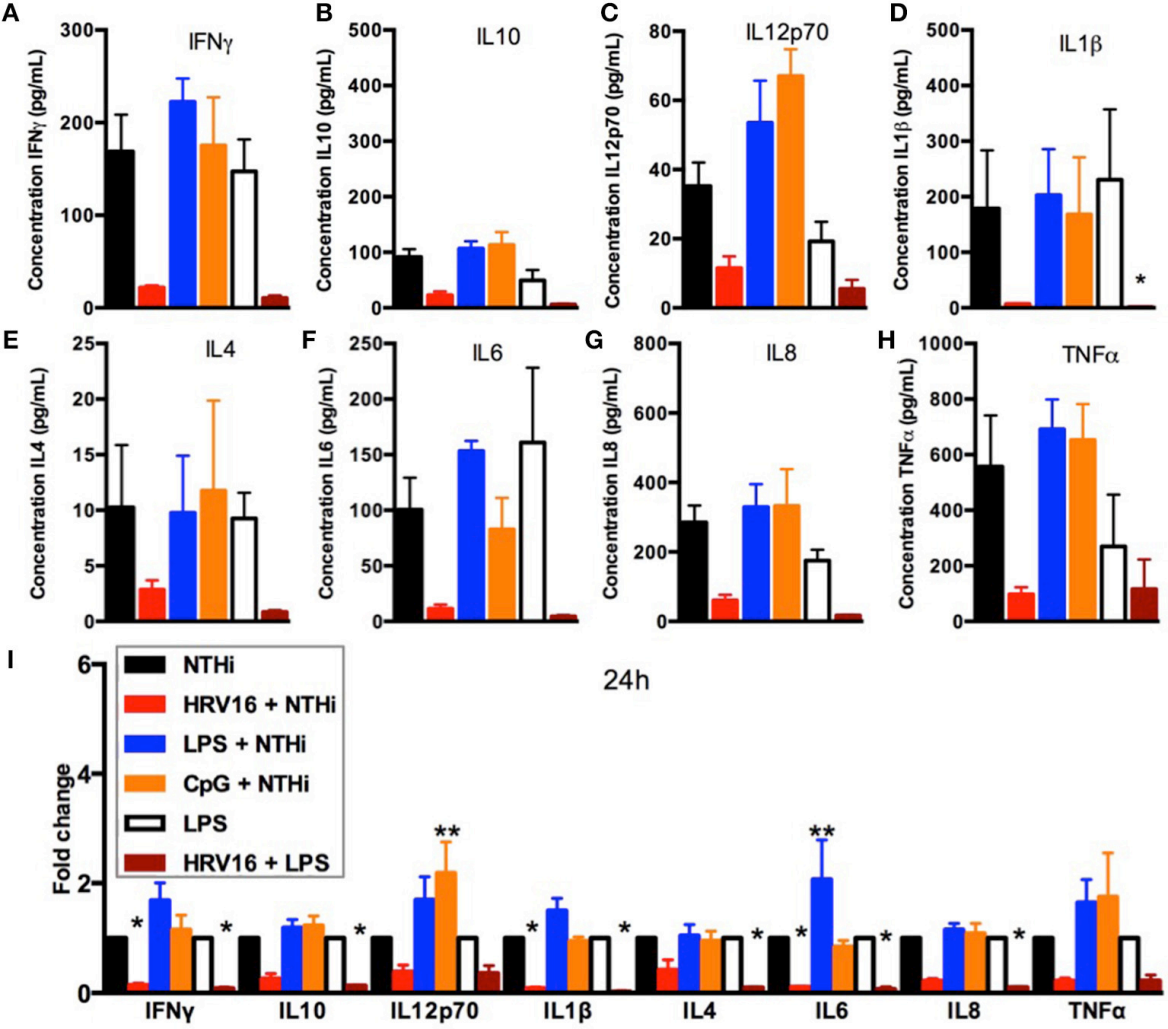
Cytokine response of human macrophages exposed to HRV16 or TLR agonists for 24 h and then challenged with NTHi or LPS. Human macrophages were exposed to HRV16 (red bars), LPS (blue bars), CpG (orange bars) or MI (black bars) for 1 h and rested overnight. Then they were exposed to NTHi or LPS for 2 h and supernatants were collected and analyzed by MSD. MSD results for **(A)** IFNγ, **(B)** IL10, **(C)** IL12p70, **(D)** IL1β, **(E)** IL4, **(F)** IL6, **(G)** IL8, **(H)** TNFα. *p < 0.05 Kruskal Wallis Test with Dunn's Post Test vs. NTHi or MI + LPS. **(I)** Relative fold changes for cytokine production in HRV16, LPS or CpG exposed human macrophages. *p < 0.05, **p < 0.01 Two Way Anova with Dunnett's Post Test vs. NTHi or MI + LPS. Error bars represent standard error of the mean (SEM). *n* = 4 independent experiments on different donors.

We next assessed if the inability to secrete cytokines in response to NTHi after HRV16 treatment was limited to viral infection or could be observed with different pre-activation triggers. Macrophages were first treated with HRV16, LPS, CpG or MI supernatants and then challenged 24 h later with NTHi for 2 h. Supernatants were collected and analyzed for cytokine secretion by the MSD technology (Figure 4). We found that HRV16 exposed macrophages were unable to secrete pro-inflammatory and regulatory cytokines in response to NTHi as seen above (Figures 4A–I). This was not observed if the cells were pre-activated with LPS or CpG (Figures 4A–I), demonstrating that the defective response to a second trigger was specific to viral pre-treatment.

### HRV16 Infection Impairs Cytokine Secretion From Human Macrophages in Response to LPS

We next analyzed if the diminished secondary response was restricted to NTHi or extended to bacterial products such as LPS that is a potent stimulator of macrophages. Macrophages were first treated with HRV16 or MI supernatants and were challenged 24 h later with LPS for 2 h. Supernatants were collected and analyzed for cytokine secretion by the MSD technology (Figure 4). There was a diminished production of all cytokines analyzed in macrophages exposed to LPS when the cells had been pre-treated with HRV16 compared to MI (Figures 4A–I). This shows that the defect caused by HRV16 extends beyond NTHi infection.

### HRV16 Impairment of Secondary Responses to NTHi Is Still Present at 48 and 72 h

**Figure 5:**
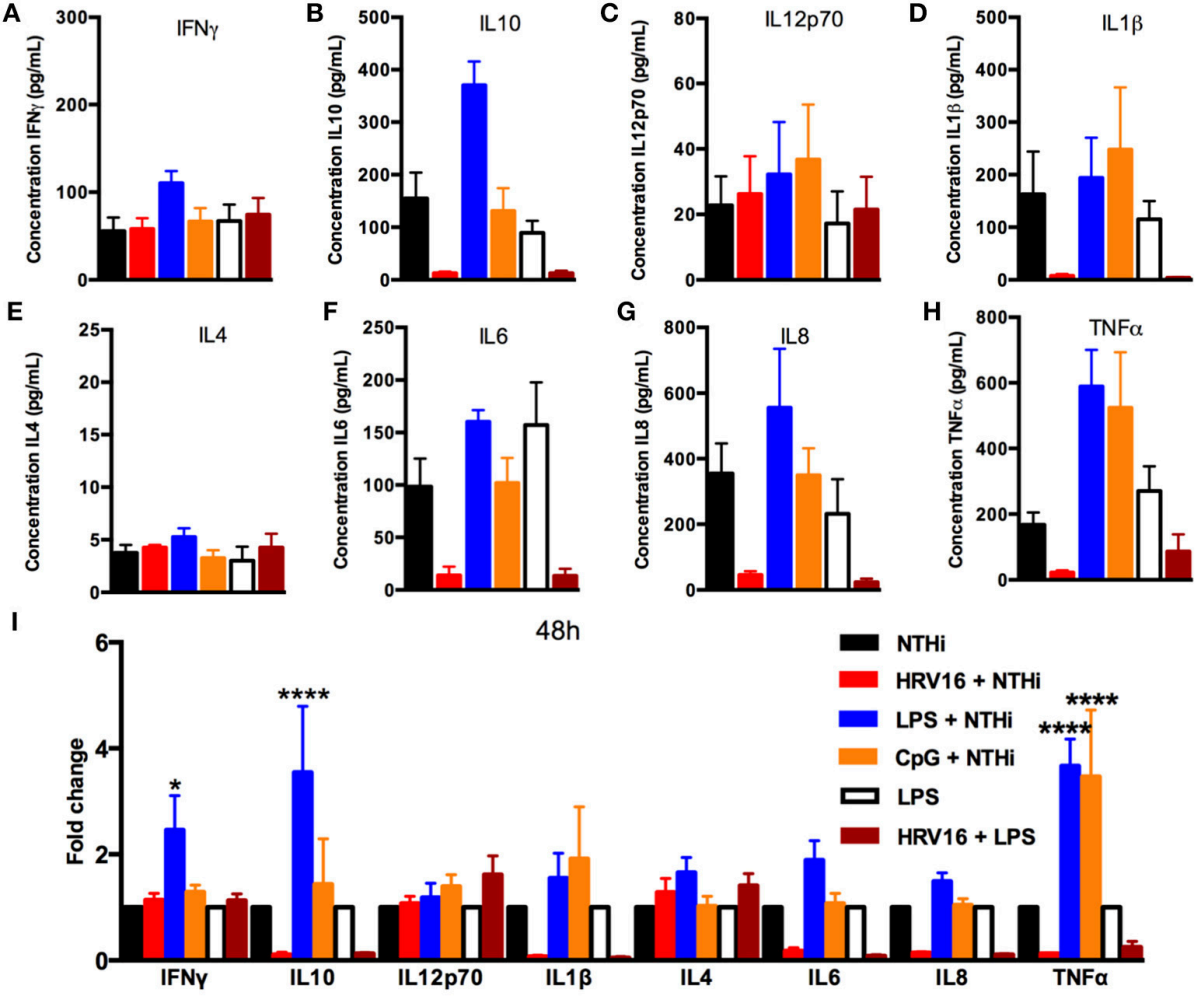
Cytokine response of human macrophages exposed to HRV16 or TLR agonists for 48 h and then challenged with NTHi or LPS. Human macrophages were exposed to HRV16 (red bars), LPS (blue bars), CpG (orange bars) or MI (black bars) for 1 h and rested for 48 h. Then they were exposed to NTHi or LPS for 2 h and supernatants were collected and analyzed by MSD. MSD results for **(A)** IFNγ, **(B)** IL10, **(C)** IL12p70, **(D)** IL1β, **(E)** IL4, **(F)** IL6, **(G)** IL8, **(H)** TNFα. **(I)** Relative fold changes for cytokine production in HRV16, LPS or CpG exposed human macrophages. *p < 0.05, ****p < 0.0001 Two Way Anova with Dunnett's Post Test vs. NTHi or MI + LPS. Error bars represent standard error of the mean (SEM). *n* = 4 independent experiments on different donors.

**Figure 6:**
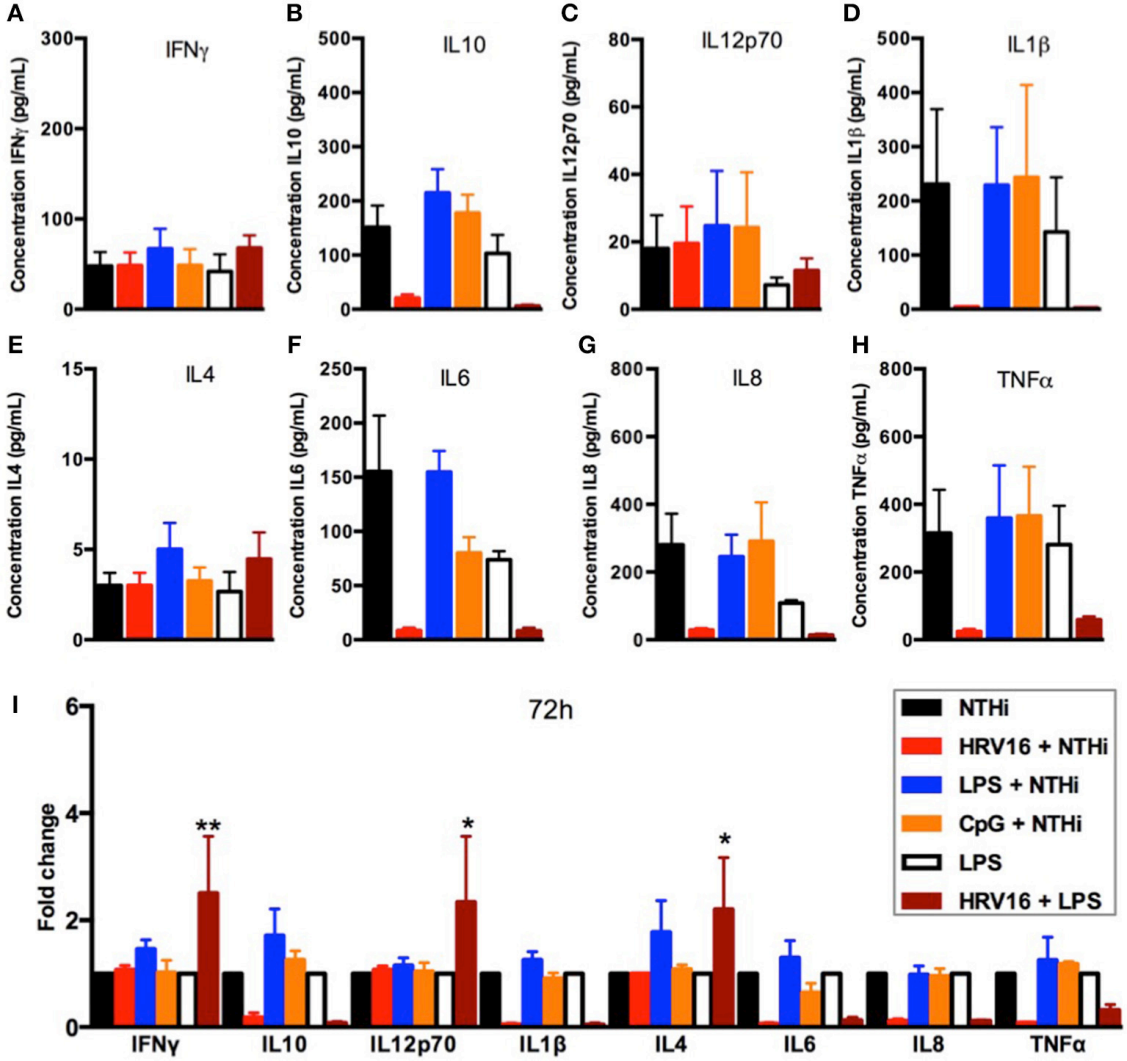
Cytokine response of human macrophages exposed to HRV16 or TLR agonists for 72 h and then challenged with NTHi or LPS. Human macrophages were exposed to HRV16 (red bars), LPS (blue bars), CpG (orange bars) or MI (black bars) for 1 h and rested for 72 h. Then they were exposed to NTHi or LPS for 2 h and supernatants were collected and analyzed by MSD. MSD results for **(A)** IFNγ, **(B)** IL10, **(C)** IL12p70, **(D)** IL1β, **(E)** IL4, **(F)** IL6, **(G)** IL8, **(H)** TNFα. **(I)** Relative fold changes for cytokine production in HRV16, LPS or CpG exposed human macrophages. *p < 0.05, **p < 0.01 Two Way Anova with Dunnett's Post Test vs. NTHi or MI + LPS. Error bars represent standard error of the mean (SEM). *n* = 4 independent experiments on different donors.

To address whether the inhibitory effect of HRV16 would last more than 24 h, macrophages were first treated with HRV16, LPS, CpG or MI supernatants and then challenged 48 h or 72 h later with NTHi for 2 h (Figures 5, 6, respectively). We found that the diminished secretory response remained at 48 and 72 h for IL10, IL6, IL8, IL1β, and TNFα (Figures 5, 6B,D,F–I). The response triggering IL4, IL12p70 and IFNγ secretion appeared to be restored at these later time points (Figures 5, 6A,C,E), but we noted that the MI controls were secreting progressively less of these cytokines at later time points (Figures 5, 6A,I). Further, if we challenged macrophages with LPS 48 or 72 h after HRV16 exposure, we found that the production of IL10, IL6, IL8, IL1β, and TNFα were still diminished (Figures 5, 6B,D,F–I). This demonstrates that macrophages still present inhibited responses toward second triggers following HRV16 exposure beyond 24 h.

**Figure 7:**
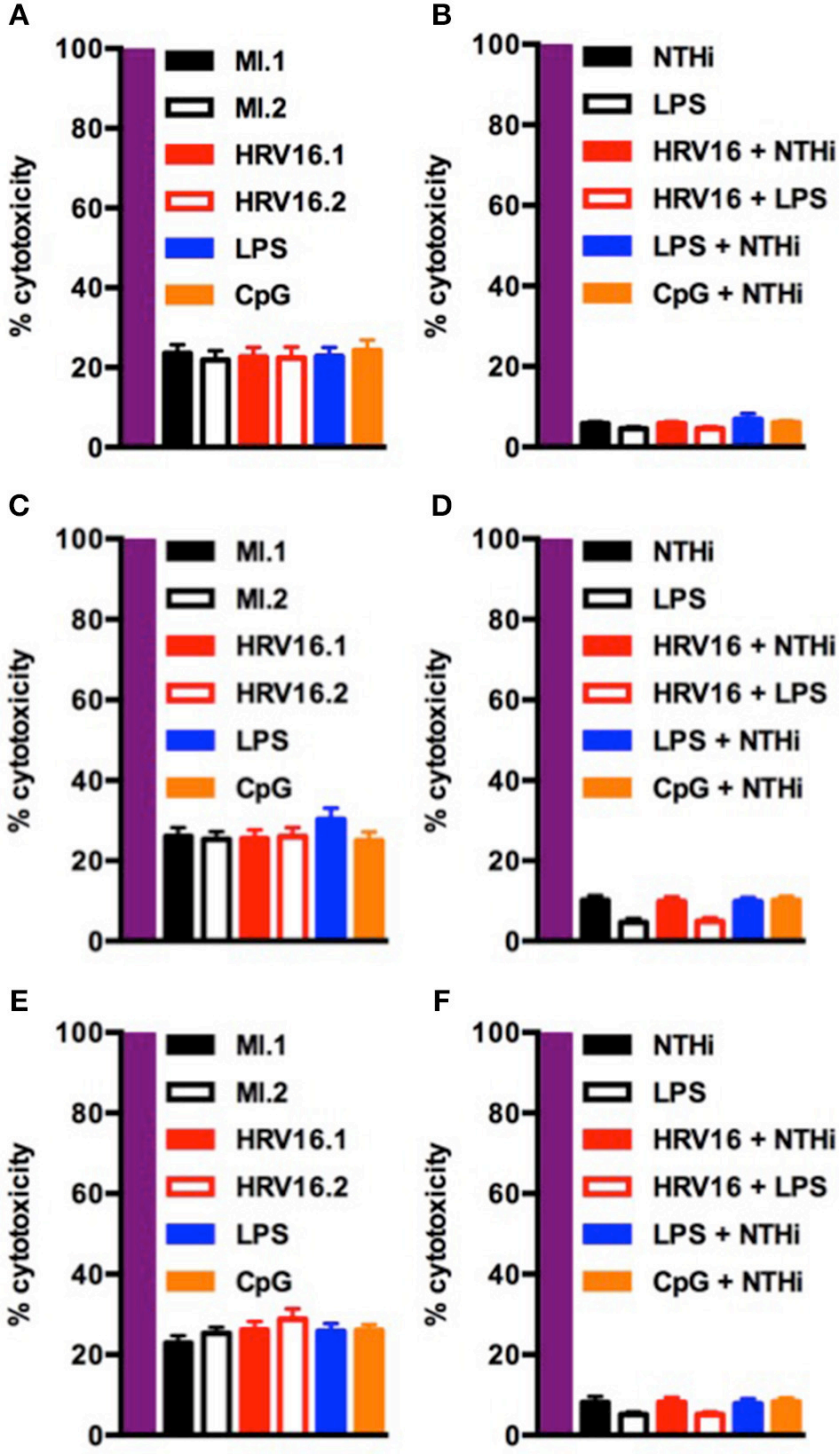
Human macrophages exposed to HRV16 do not display increased cytotoxicity. Human macrophages were exposed to HRV16 (red bars), LPS (blue bars), CpG (orange bars) or MI (black bars) for 1 h and rested overnight, for 48 or 72 h. Then they were exposed to NTHi or LPS for 2 h. Supernatants were collected and analyzed by an LDH assay. LDH results for **(A)** 24 h, **(B)** 24 h + 2 h NTHi or LPS, **(C)** 48 h, **(D)** 48 h + 2 h NTHi or LPS, **(E)** 72 h, **(F)** 72 h + 2 h NTHi or LPS. All results are expressed relative to total lysis (purple bars). *n* = 4 independent experiments on different donors.

Finally, to confirm that the failure of HRV16 exposed macrophages to secrete cytokines was not due to enhanced cell death, we performed a lactate dehydrogenase (LDH) using the presence of this enzyme in cell supernatants to monitor cell permeability and death, as compared with the activity measured after total cell lysis (Figure 7). We observed no increase in cytotoxicity in HRV16 exposed macrophages +/– NTHi or LPS, compared to control conditions over 72 h (Figure 7).

Together, these results demonstrate that HRV16 exposed macrophages are unable to mount an efficient response toward secondary targets, in this case bacteria or LPS, and that the defective response persists in part for at least 72 h.

## Discussion

In this study, we demonstrate that macrophages respond to HRV16 by secreting inflammatory cytokines, but this response is altered upon secondary challenge with live bacteria or bacterial compound such as LPS. This is important, because HRV is routinely isolated at COPD exacerbations (20, 21) and thought to contribute to the dysregulated microbiome seen in these patients (6).

We found that macrophages exposed to HRV16 produced elevated levels of cytokines compared to uninfected control conditions. We detected robust production of TNFα, IL10 and IL1β in response to HRV16. Modest increases in IFNγ, IL4, IL6, and IL12p70 were also observed. Furthermore, in these cells comparable secretion of most cytokines, except IL10, was noted with CpG activation. LPS challenge of these cells also resulted in cytokine secretion in a range higher or similar to that obtained with HRV16 challenge. These results fit with other studies showing that HRV leads to a robust cytokine response (22–28) and specifically in monocytes/macrophages (29–32). HRV is known to cause robust IFNα and IFNβ production (33, 34). In our experiments, we could not detect IFNβ secretion despite elevated mRNA levels (data not shown). In agreement with our observations, other studies have shown no detectable IFNβ secretion in response to HRV (26, 35, 36). It has also been reported that different HRV strains induce different cytokine responses (37, 38) with clinical strains inducing more release of IL6, IP10, IFNγ and IFNβ (28).

Our second major finding was that macrophages infected with HRV16 and subsequently with NTHi or LPS produce less pro-inflammatory and regulatory cytokines compared to control cells. In addition, this phenotype lasts for at least 72 h toward the majority of cytokines tested. Importantly, the diminished cytokine responses in HRV16 exposed macrophages were not due to increased cytotoxicity. Of note, our observations do not indicate any trend toward a macrophage polarization, but rather a “paralyzed” phenotype that was not mimicked when cells were pre-activated with agonists like LPS or CpG instead of the virus. This was observed even toward those cytokines that showed modest increases in response to HRV16. The same altered response was not seen with HRV16^UV^, suggesting that it is specific to live HRV16. How the virus precisely regulates the cytokine secretion in response to a secondary challenge, however, still requires further investigation. This is critical, because it has been shown that viruses from clade A of the HRV group, including HRV16, are frequently associated with severe COPD exacerbations (39). They are associated with increased possibility of bacterial detection and postulated to be related to secondary effects on the outgrowth of bacteria.

How HRV affects the cytokine response toward bacteria has received limited attention in macrophages. Lung macrophages challenged with HRV showed reduced IL8 and TNFα production in response to LPS and LTA (15). In epithelial cells, HRV and then NTHi exposure led to decreased production of IL8 (40). Combined with our data, these results suggest that HRV can specifically shutdown macrophage responses and cytokine secretion in response to bacterial infection. Our *in vitro* data is not reflective of the entire lung environment where a complete microbiome is present, but our findings could nevertheless contribute to explain how HRV hijacks macrophage functions within the lung and potentially explain why co-infections are increasingly documented in COPD exacerbations.

## Author Contributions

FN, NK, and GM conceived and designed the study. JJ, KA-G, FH, and EB designed and performed experiments and collected data. GM, DC, NK, and FN contributed to design experiments. All authors analyzed the data. JJ, EB, GM, DC, NK, and FN contributed to writing the manuscript.

### Conflict of Interest Statement

NK, DC, GM, and EB are employed by the commercial company “AstraZeneca” and AstraZeneca supported salaries for JJ and KA-G as part of a collaborative grant with FN. EB is a fellow of the AstraZeneca postdoc programme. The remaining author declares that the research was conducted in the absence of any commercial or financial relationships that could be construed as a potential conflict of interest. The handling Editor declared a shared affiliation, though no other collaboration, with one of the authors FN.
